# Combatting Meta-Ignorance in Science Through Dialogs with Diverse Experts

**DOI:** 10.1007/s42113-026-00316-5

**Published:** 2026-09-15

**Authors:** Michael Smithson

**Affiliations:** School of Medicine and Psychology, The Australian National University, Canberra, A.C.T Australia

**Keywords:** Ignorance, Illusion, Meta-ignorance, Scientific understanding, Misbelief

## Abstract

In their provocative paper, Shiffrin, Stigler, and Keil (SSK) declare that scientists have illusions of understanding the phenomena they study “more deeply” than they really do. In this comment I grant their claim a degree of truth and focus on the nature of illusions of understanding. These are a form of meta-ignorance (not knowing what you don’t know). This comment briefly describes the psychology behind meta-ignorance and how it may be combatted. A crucial property of meta-ignorance is that it is impossible for us to attribute meta-ignorance to our here-and-now selves. It can only be detected by others, but probably not by those who share our views and expertise. Instead, it will most likely be detected by those who think differently from us. Thus, ridding ourselves of meta-ignorance about our scientific understanding requires dialogs with scholars whose backgrounds, expertise, and/or orientations differ from our own.

In their provocative paper, Shiffrin, Stigler, and Keil (SSK) declare that scientists have illusions of understanding the phenomena they study “more deeply” than they really do. While there is a substantial empirical literature regarding laypeople’s overestimations of how much they know (e.g., the Dunning-Kruger effect), less is known about the extent to which scientists do so. There is some evidence that experts overestimate and overclaim knowledge, although these tendencies may be strongest for those who have only introductory-level education on a topic (Atir & Dunning, 2025). Nevertheless, I’ll grant SSK’s claim some degree of truth. In this commentary I will discuss factors that may be amplifying the problem and suggest ways of combatting it.

To begin, illusions of understanding are a form of meta-ignorance (not knowing what you don’t know). SSK are saying that scientists don’t know that they don’t understand their subject-matter deeply. We need a psychology of ignorance here. There are two kinds of meta-ignorance: misbelief and unconsidered ignorance. Misbelief has four kinds: belief in a truth but without adequate justification, mistaken agnosticism about a truth, disbelief in a truth, and belief in a falsehood. Unconsidered ignorance has two kinds: never having considered a proposition and being unaware that a proposition exists (Smithson, 2026).

It should be clear that some kinds of meta-ignorance are more easily overcome than others; more on that shortly. But the crucial property of meta-ignorance is that we cannot attribute it to our here-and-now selves. Self-attributions such as “I have a false belief” or “I’m unaware that X exists” are nonsensical. Only other people can attribute meta-ignorance to any of us, and we can only self-attribute it retrospectively.

Two consequences follow. First, because there are several kinds of ignorance that we can only attribute to others, we may routinely attribute more ignorance to others than to ourselves. This will likely amplify our knowledge-overestimation and overplacement tendencies and probably partly account for them (Smithson, 2026).

Second, merely thinking harder is unlikely to rid us of meta-ignorance. Instead, dialogs are crucial here. Even explaining our interpretations of concepts or phenomena to others and receiving their interpretations can uncover our own meta-ignorance. SSK do not mention that in the Rozenblit-Keil study after participants had attempted to explain how a simple mechanism (e.g., a flush toilet) works and after reading an expert’s explanation, their ratings of their degree of understanding plunged. Kevin Dunbar’s (1999) field studies of how scientists work underscored this insight. Instead of solitary “Eureka” moments, most discoveries and intense reasoning occurred during weekly lab discussions.

Dialogs are essential for overcoming specific kinds of meta-ignorance. Further solitary investigations and critical thinking may reveal justification for a true belief that lacks it or correct a mistaken agnosticism. However, overcoming misbelief (and the confirmation bias that accompanies it) more likely will require constructive and extensive dialogs with scientists or scholars. Scholars who share our views and expertise also will share our meta-ignorance. Thus, it will most likely be detected in dialogs with those who think differently from us.

Likewise, dialogs with scholars from outside our silos are the most effective antidote to unconsidered meta-ignorance. For instance, “ipsative” (compositional) measurement fell out of favor in psychology during the 1960’s because it induces dependencies among observations that familiar statistical techniques cannot handle. For decades the consensus was that there was no way to transform compositional data to noncompositional (and analyzable) forms. Unbeknown to psychologists, such transformations were worked out by statisticians in the early 1980’s and thereafter widely applied in geology and biology. It took four decades for it to be imported into psychology (Smithson & Broomell, 2024).

Also essential are incentives. Incentives to overcome meta-ignorance are almost absent in academia. Current incentive structures do not reward self-attributions of ignorance anyway, especially when such attributions are to be communicated to others (e.g., teachers, grant funding agencies, promotion committees). Consequently, we do not develop habits of reflecting and communicating deeply on what we don’t know or understand (how often have any of us delivered conference talks whose *main topic* was what we don’t know? ). Instead, research funders reward researchers who claim (and sometimes believe) they know what they’re going to find before they’ve done the research.

One of the best antidotes to nearly any variety of ignorance is curiosity, plus the time, freedom, and resources to pursue the investigations driven by it. But current reward structures in academia discourage curiosity-driven research. These structures reward those who orient their research to the latest priorities of research funders, to what is likely to garner high citation rates, and to rapid publication.

In the longer term, the incentive structures in science education, research funding, and career security and advancement need changing. They need to reward intellectual work practices that involve describing, analyzing, and communicating one’s ignorance and pursuing curiosity-driven research arising from awareness about the scope and depth of what one doesn’t know.

But in the shorter term, the chief remedies for excavating and removing meta-ignorance are constructive critical dialogs with colleagues from diverse backgrounds and perspectives. The path to overcoming our meta-ignorance about our scientific understanding isn’t through thinking harder. It’s through thinking together.

## Data Availability

No datasets were generated or analysed during the current study.
